# Supplementary material for: Quantitative imaging of Caenorhabditis elegans dauer larvae during cryptobiotic transition
Source: Biophys J. 2022 Feb 19;121(7):1219–29. doi: 10.1016/j.bpj.2022.02.031 (PMC9034246; doi:10.1016/j.bpj.2022.02.031)
Supplement: Document S1. Figures S1–S6 [file mmc1.pdf]

Biophysical Journal, Volume *121*

Supplemental information

Quantitative imaging of *Caenorhabditis elegans* dauer larvae during cryptobiotic transition

Kyoohyun Kim, Vamshidhar R. Gade, Teymuras V. Kurzchalia, and Jochen Guck

Supplementary Information for
Quantitative imaging of *Caenorhabditis elegans* dauer larvae during cryptobiotic transition

Kyoohyun Kim[1,2,†], Vamshidhar R. Gade[3,4,†], Teymuras V. Kurzchalia[3,*], and Jochen Guck[1,2,*]

[1]Biotechnology Center, Center for Molecular and Cellular Bioengineering, Technische Universität Dresden, 01307 Dresden, Germany

[2]Max Planck Institute for the Science of Light & Max-Planck-Zentrum für Physik und Medizin, 91058 Erlangen, Germany

[3]Max Planck Institute of Molecular Cell Biology and Genetics, 01307 Dresden, Germany

[4]Present address: Institute of Biochemistry, ETH Zürich, 8093 Zürich, Switzerland

[†]K.K. and V.G. contributed equally to this work.

[*]To whom correspondence may be addressed.

Email: kurzchalia@mpi-cbg.de or jochen.guck@mpl.mpg.de

This PDF file includes:

Figures S1 to S6
Legends for Movies S1 to S4
SI References

Other supplementary materials for this manuscript include the following:

Movies S1 to S4

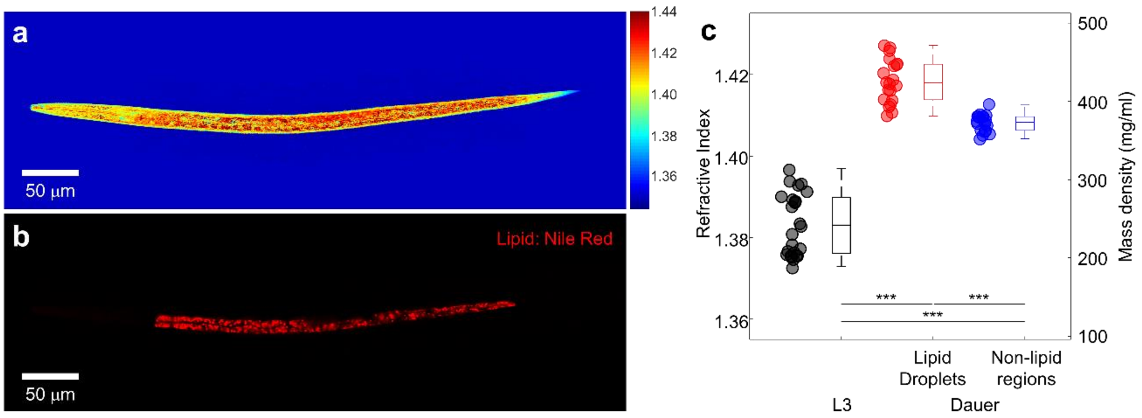

Supplementary Figure 1. Correlation between refractive index (RI) tomograms and epi-fluorescence images of lipid droplets in dauer larvae. (a) Central cross-sectional slice through an RI tomogram of a typical dauer larva. Color scale shows RI. (b) Epi-fluorescence image of the same dauer larva in which the lipid content is stained with Nile Red. (c) Mean RI of larvae at the L3 stage and lipid droplets and non-lipid regions in the Nile Red-stained dauer larvae. The RI distribution of larvae at the L3 stage is reused from Figure 2e. Please note that the absolute values of both lipid droplets and non-lipid regions are slightly higher than the overall RI values reported in Figure 2b and e. This could either be due to a slight batch-to-batch variability in the dauers or to a slight increase in the mass density due to the Nile Red staining. The numbers of L3 and dauer larvae measured are N = 25 and 20, respectively.

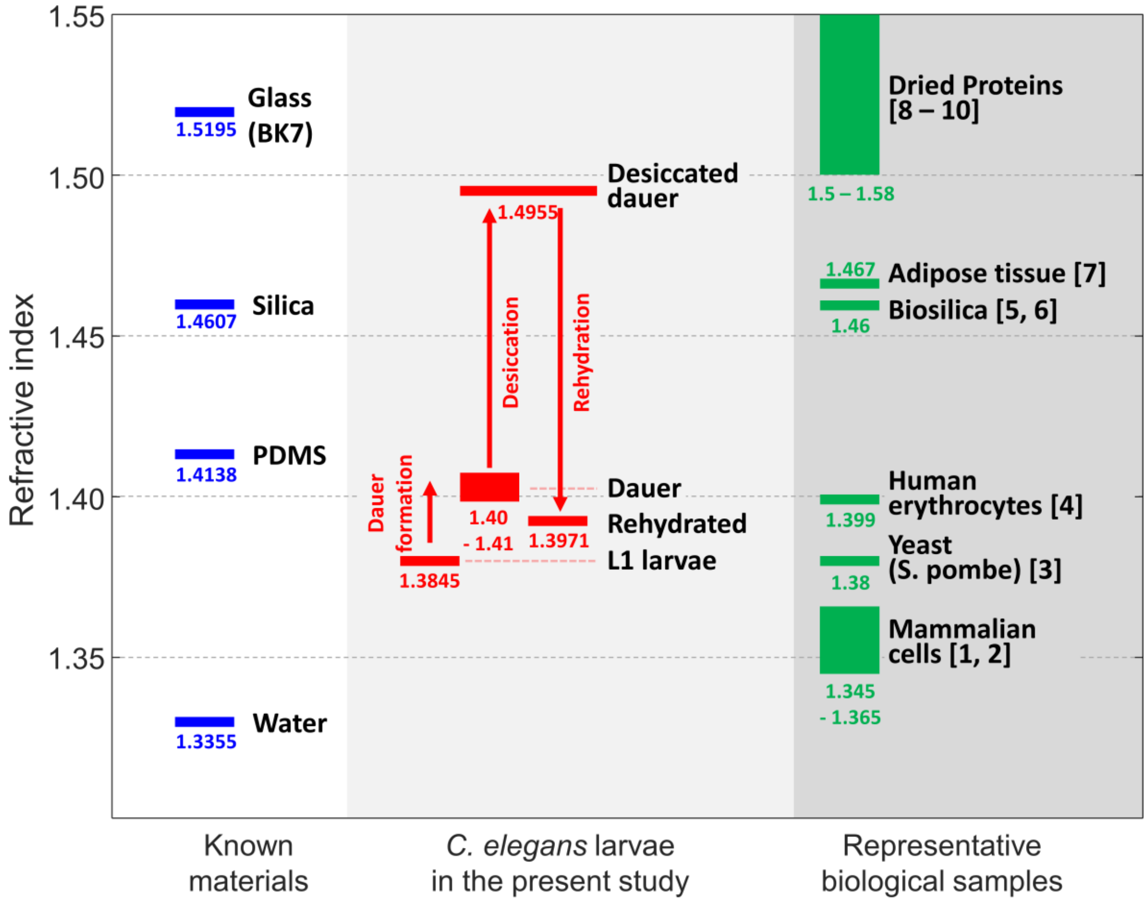

Supplementary Figure 2. Graphical summary on the measured refractive index (RI) value of *C. elegans* larvae in the present study (center) compared to the RI of known inanimate materials (left) and representative biological samples (right).

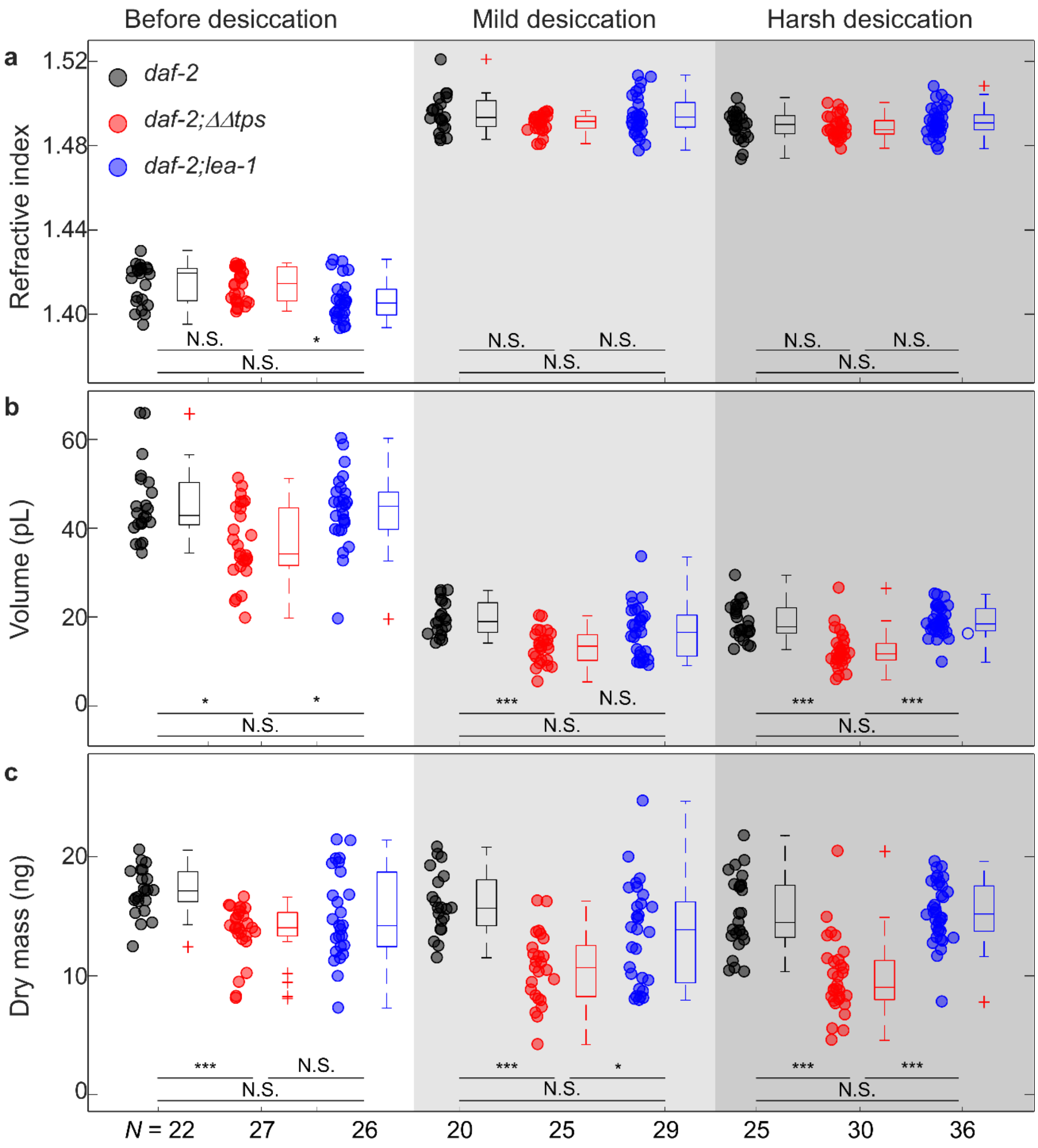

Supplementary Figure 3. Quantitative characterization of wild type (black), trehalose deletion mutant (red), and LEA-1 deletion mutant (blue) dauer larvae in different conditions. (a) The mean refractive index, (b) volume, and (c) dry mass of the dauer larvae.

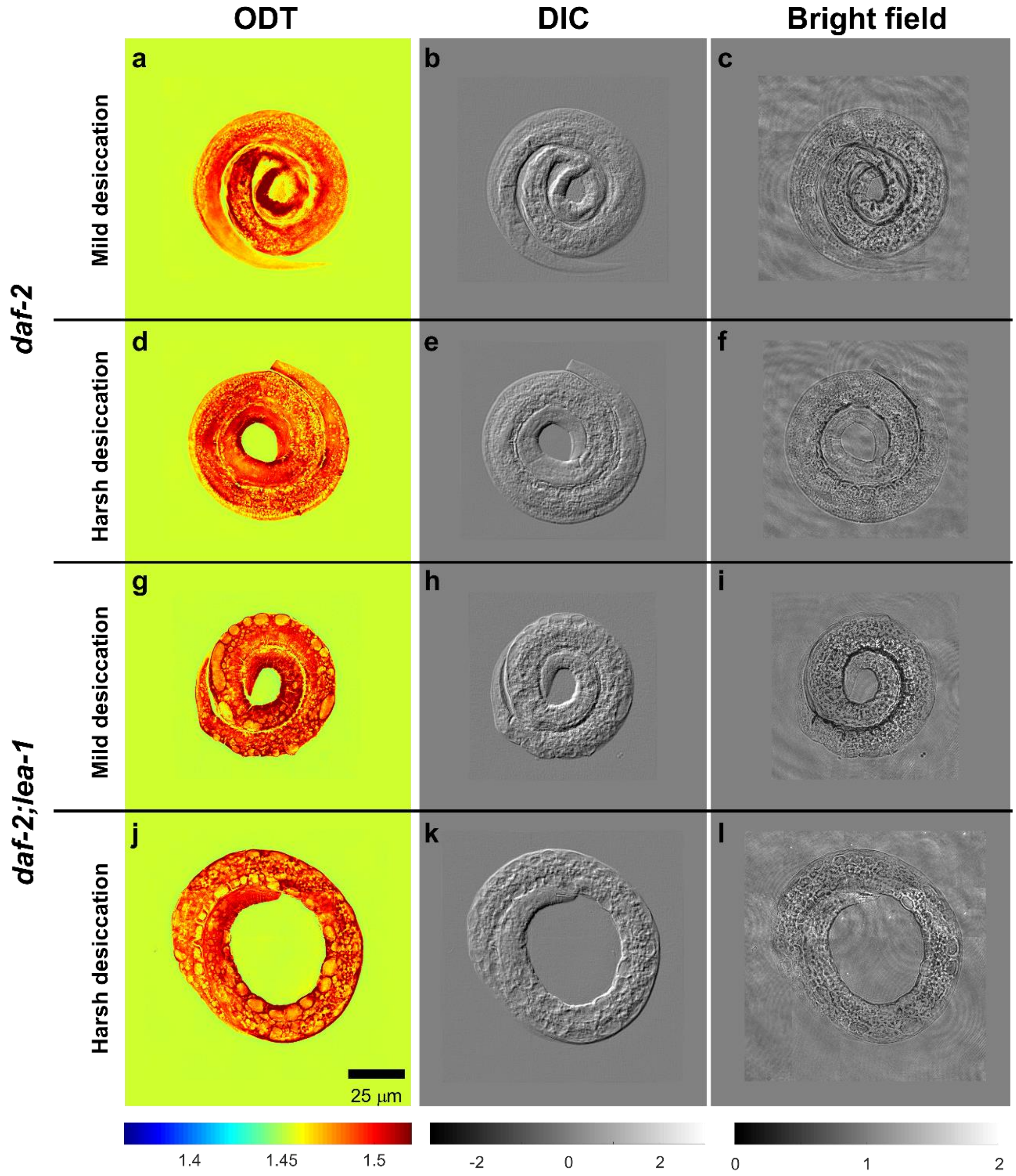

Supplementary Figure 4. Comparison of images acquired by different microscopy modalities. (a, d, g, j) Typical central cross-sectional slices through RI tomograms, (b, e, h, k) emulated differential interference contrast (DIC) images, and (c, f, i, l) bright field images of *C. elegans* dauer larvae. (a – c) represent wild type after mild (98% RH) desiccation, (d – f) wild type after harsh (60% RH) desiccation, (g – i) lea-1 deletion mutants *daf-2;lea-1* after mild desiccation, and (j – l) *daf-2;lea-1* after harsh desiccation. Color scale shows RI. Grey scales show light intensity in the arbitrary unit.

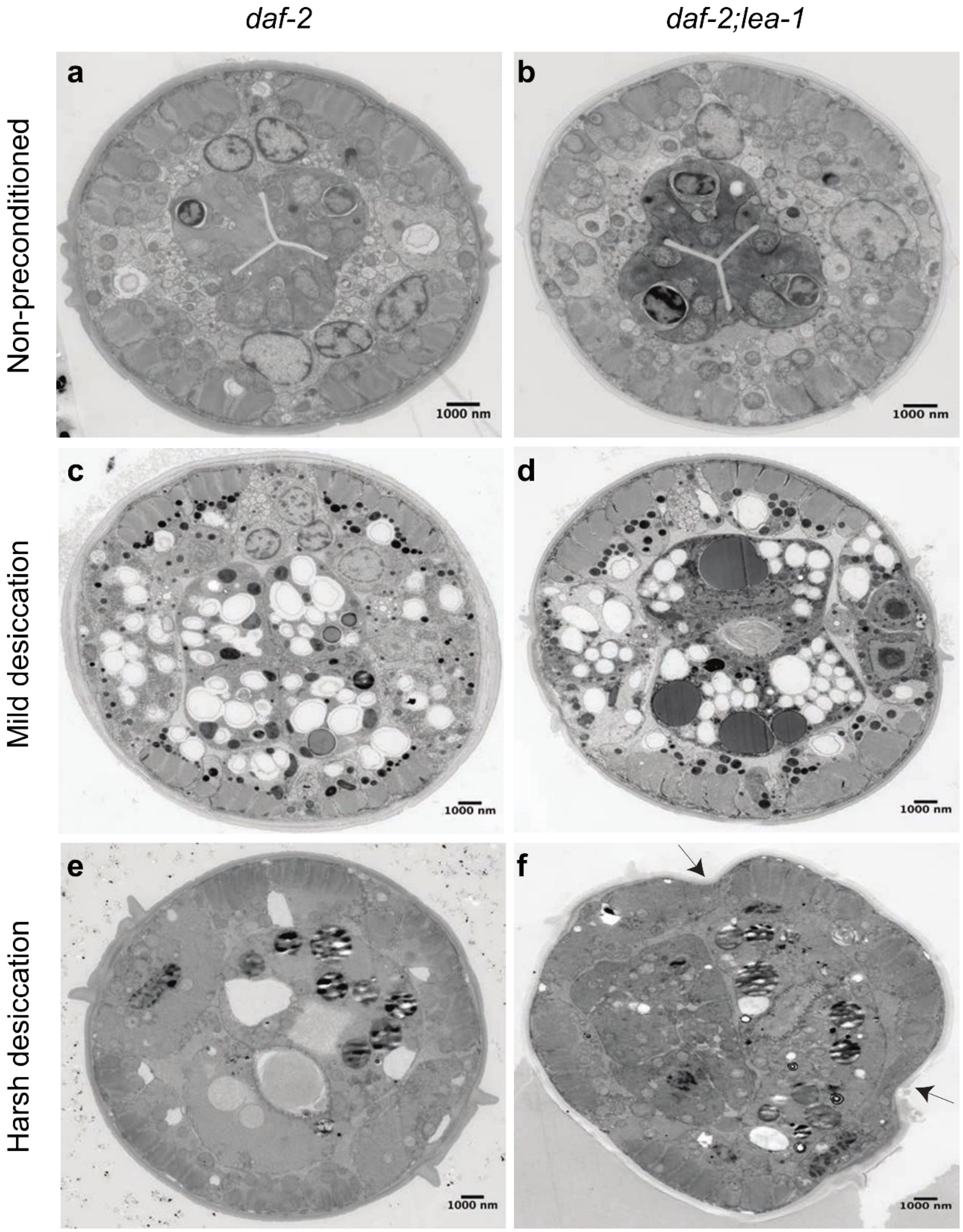

Supplementary Figure 5. Electron micrographs of (a, c, e) wild type (*daf-2*) and (b, d, f) *lea-1* deletion mutant (*daf-2;lea-1*) in (a, b) the non-preconditioned, (c, d) mild desiccation, and (e, f) harsh desiccation conditions. The arrows in f indicate the shrinkage of the desiccated dauer larvae of *lea-1* deletion mutant.

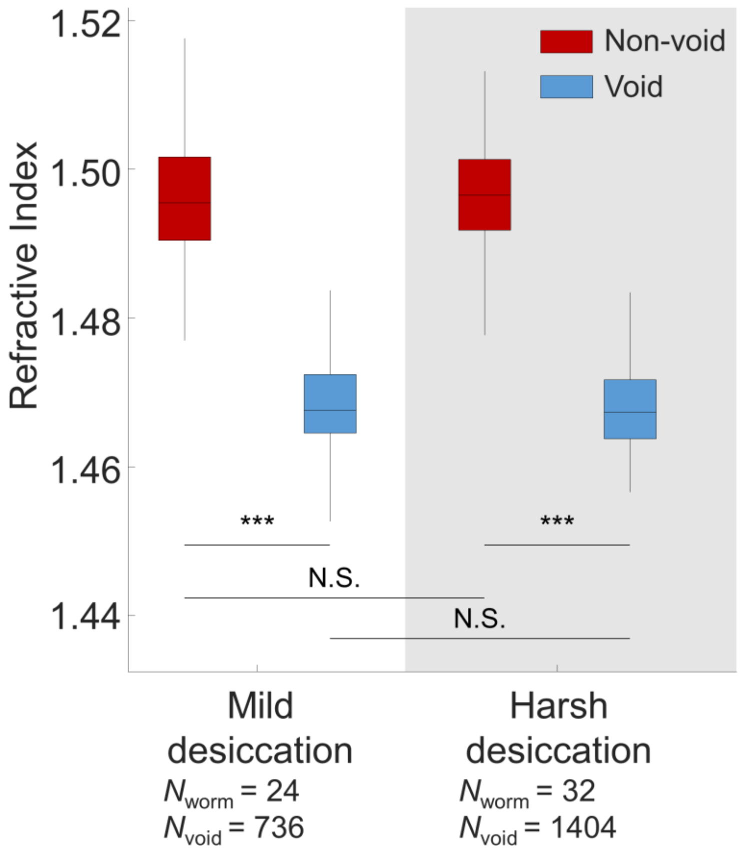

Supplementary Figure 6. Refractive index in void regions and their periphery of *lea-1* deletion mutant during mild and harsh desiccation conditions.

Supplementary Movie 1. Visualization of the RI tomogram and rendered isosurface of a typical *C. elegans* larva at the L3 stage.

Supplementary Movie 2. Visualization of the RI tomogram and rendered isosurface of a typical *C. elegans* dauer larva of controls after harsh desiccation (60% RH).

Supplementary Movie 3. Visualization of the RI tomogram and rendered isosurface of a typical *C. elegans* dauer larva of trehalose deletion mutants *daf-2;ΔΔtps* after harsh desiccation (60% RH).

Supplementary Movie 4. Visualization of the RI tomogram and rendered isosurface of a typical *C. elegans* larva of *lea-1* deletion mutants *daf-2;lea-1* after harsh desiccation (60% RH).

Supplementary References

1. P. Y. Liu, et al., Cell Refractive Index for Cell Biology and Disease Diagnosis: Past, Present and Future. *Lab Chip* **16**, 634–644 (2015).
2. M. Schürmann, et al., Three-dimensional correlative single-cell imaging utilizing fluorescence and refractive index tomography. *J. Biophotonics* **11**, e201700145 (2018).
3. S. Abuhattum, et al., Intracellular Mass Density Increase Is Accompanying but Not Sufficient for Stiffening and Growth Arrest of Yeast Cells. *Front. Phys.* **6**, 131 (2018).
4. Y. Park, et al., Refractive index maps and membrane dynamics of human red blood cells parasitized by Plasmodium falciparum. *Proc. Natl. Acad. Sci.* **105**, 13730–13735 (2008).
5. J. Aizenberg, V. C. Sundar, A. D. Yablon, J. C. Weaver, G. Chen, Biological glass fibers: Correlation between optical and structural properties. *Proc. Natl. Acad. Sci.* **101**, 3358–3363 (2004).
6. J. M. Soto, J. A. Rodrigo, T. Alieva, Label-free quantitative 3D tomographic imaging for partially coherent light microscopy. *Opt. Express* **25**, 15699–15712 (2017).
7. I. Y. Yanina, E. N. Lazareva, V. V. Tuchin, Refractive index of adipose tissue and lipid droplet measured in wide spectral and temperature ranges. *Appl. Opt.* **57**, 4839–4848 (2018).
8. Arwin, H. Optical Properties of Thin Layers of Bovine Serum Albumin, γ-Globulin, and Hemoglobin. *Appl. Spectrosc.* **40**, 313–318 (1986).
9. Wang, X. J., Milner, T. E., Chang, M. C. & Nelson, J. S. Group refractive index measurement of dry and hydrated type I collagen films using optical low-coherence reflectometry. *J. Biomed. Opt.* **1**, 212–216 (1996).
10. Bucciarelli, A. *et al.* A comparative study of the refractive index of silk protein thin films towards biomaterial based optical devices. *Opt. Mater. (Amst).* **78**, 407–414 (2018).
